# A systematic review of the adolescent‐directed marketing strategies of transnational fast food companies in low‐ and middle‐income countries

**DOI:** 10.1002/osp4.676

**Published:** 2023-06-19

**Authors:** Elijah Bankole, Neil Harris, Shannon Rutherford, Nicola Wiseman

**Affiliations:** ^1^ Public Health School of Medicine and Dentistry Griffith University Gold Coast Queensland Australia

**Keywords:** adolescents, fast food, marketing, obesity

## Abstract

**Introduction:**

Fast food consumption is associated with excessive intake of energy‐dense foods; a major determinant of childhood obesity. The lack of data on the marketing strategies used to promote fast food to adolescents in low and middle‐income countries (LMICs) acts as a barrier to global efforts to reduce the marketing of unhealthy foods to young people around the world.

**Objectives:**

This systematic review aimed to identify the adolescent‐directed marketing strategies of transnational fast food corporations in LMICs.

**Methods:**

A systematic search of eight scientific databases (PubMed, CINAHL, Medline, Embase, ProQuest, PsycInfo, Scopus and Google Scholar) was conducted. Following PRISMA guidelines, primary research articles written in English were included if they were published between 1 January 2010 and 30 December 2022, and reported any adolescent‐directed marketing activity undertaken by a transnational fast food company operating in a LMIC. Articles were excluded if they were not peer reviewed. The quality of the included articles was assessed using a condensed version of the Consolidated Criteria for Reporting Qualitative Research tool.

**Results:**

Twelve articles met the eligibility criteria and were included in this review. A narrative synthesis of these articles revealed that the most documented strategies used to promote fast food to adolescents in LMICs were the use of incentives or premium offers, product appeals, promotional characters and brand familiarity. These strategies were mostly observed on social media, suggesting that there are serious concerns about adolescent exposure to fast food via social media in developing settings, especially as contextual differences in the nature of such marketing were identified.

**Discussion:**

The promotion of fast food to adolescents in LMICs is contextual in nature, with the nature of marketing strategies employed by transnational fast food corporations varying greatly across cultural and socio‐economic contexts. These findings are crucial for the development of guidelines and regulations restricting the marketing of fast food to adolescents in lower income settings, contributing to global efforts to reduce adolescent exposure to unhealthy food promotion.

## Graphical abstract

Fast food consumption is associated with excessive intake of energy‐dense foods; a major determinant of childhood obesity. The lack of data on the marketing strategies used to promote such foods to adolescents in low and middle‐income countries (LMICs) acts as a barrier to global efforts to reduce the marketing of unhealthy foods to young people around the world. This systematic review sought to reveal the common marketing strategies used by these corporations to promote fast food to the adolescents in these regions. The findings of this review are therefore crucial for the development of guidelines and regulations restricting the marketing of fast food to these adolescents, contributing to the efforts aimed at forestalling the obesity epidemic in the developing world.

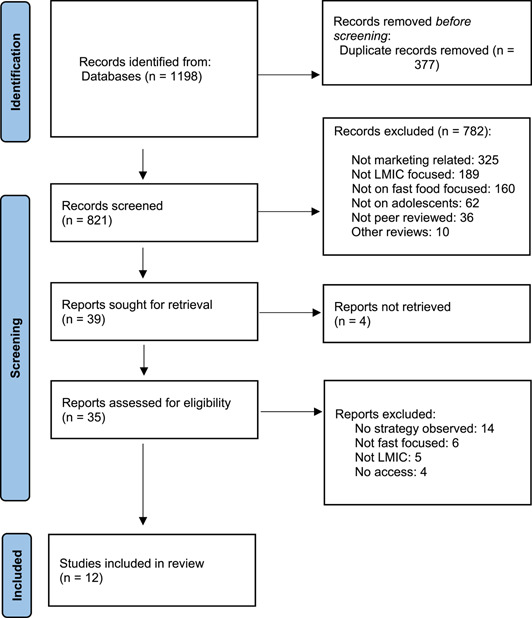

## 1 INTRODUCTION

A nutrition transition toward more energy‐dense, processed foods is in advanced stages in many low and middle‐income countries (LMICs) across South‐east Asia, Central America and sub‐Saharan Africa.^1^, ^2^ The most evident impact of this transition are the rising rates of overweight and obesity, with grave implications for population health.^2^, ^3^, ^4^, ^5^ Since 2000, the number of overweight children under 5 has increased by nearly 24% in Africa while almost half of the children under 5 who were overweight or obese in 2019 lived in Asia.^6^ These alarming increases are predominantly driven by dietary changes, with food now increasingly processed, affordable, and marketed to children worldwide.^7^

Fast food in particular, has become a “normal” feature of the food environment in developing countries, with its increased consumption recognized as a major contributor to the rising prevalence of obesity among children in LMICs.^8^ Fast food consumption is associated with excessive intake of energy‐dense foods which, along with reduced physical activity, is the major determinant of childhood obesity.^9^ Researchers have also reported positive associations between the frequency of fast food consumption and obesity,^10^ higher energy intake,^11^ and poorer diet quality^12^ among both adolescents and adults. As a result, the aggressive promotion of fast food to adolescents using techniques well‐known to appeal to their age group represents a significant public health problem.^13^ Of greater implication for global health, is the industry's shift in focus from high income countries' (HIC) markets to the new and emerging markets of LMICs.^14^

In recent times, transnational fast food corporations have been forced to focus on global expansion as a rapid growth strategy due to the increasingly competitive and health conscious nature of the market in most HICs.^14^, ^15^, ^16^ Further, with 85% of the world's population living in LMICs, and per capita wealth rising rapidly in middle income countries, this population constitutes a significant market base.^17^ Consequently, these companies have rapidly increased their presence in LMICs to a similar, or even greater extent than in high‐income countries, with countries like South Africa now hosting more KFC outlets (900) than Australia (653).^18^ Such rapid penetration, which has seen the fast food industry become major drivers of the nutrition transition occurring in LMICs, is widely attributed to foreign investment and mass marketing campaigns.^1^, ^19^, ^20^

As with alcohol and tobacco, adolescents as defined by WHO as children aged 9–19, are the prime marketing target of fast food companies, with research showing that the human brain is only able to make decisions that control primeval emotional responses by the early 20s.^21^ Further, food marketing generally aims to develop in consumers the habit of consuming the marketed product regularly.^22^ Therefore, adolescents remain the primary target of unhealthy food promotion in LMICs, as targeting this age group ensures that consumption habits are changed over the long term and future profits are guaranteed.^15^ To this end, food companies, particularly fast food brands, employ marketing strategies demonstrated to influence adolescents' food choices, with the most obvious consequence being an increase in consumption leading to weight gain within this population.^8^, ^23^

Across the globe, these strategies vary, and several researchers have explored the extent and nature of the diverse marketing techniques used by fast food brands, with Truman and Elliott^24^ recently describing common categories of adolescent‐targeted marketing strategies. For example, fast food brands often use sports‐themed marketing campaigns and promotions to appeal to adolescents.^25^, ^26^, ^27^ Other common techniques used to promote fast food to adolescents include the use of online advergames,^28^, ^29^ celebrity endorsements,^30^, ^31^ meal bundles,^32^ and price discounts advertised via television and other traditional platforms like print media and point‐of‐sale.^33^ In recent times however, children are increasingly exposed to fast food promotion through digital avenues.^34^

According to a recent study, more than half of the total marketing budget of multinational food companies is allocated toward online marketing, which now spans across digital media such as food company websites, applications and social media platforms.^34^ Worryingly, this avenue for food brand exposure is targeted predominantly at children and adolescents due to their habitual engagement with online media,^32^ with one in three Internet users around the world below the age of 18.^35^ Adolescents are also prime targets due to their increasing autonomy and spending power; their influence on household purchases; and their role in setting and following trends.^36^, ^37^ Using digital mediums, fast food companies are now able to capitalize on these qualities while maximizing impact through personalized marketing and behavioral tracking techniques.^32^ Consequently, adolescents have been shown to be hugely influenced by this new form of marketing.^38^, ^39^

Critically, regardless of the marketing channel, there is limited research on the marketing strategies used by fast food companies in LMICs, where the obesity epidemic is rapidly spreading.^6^, ^8^, ^40^ This is hugely concerning as food marketers use various techniques to appeal to different populations, with recent research revealing differences in the strategies being used in LMICs compared to HICs.^40^, ^41^ For example, Bragg and her colleagues observed that transnational fast food corporations promoted fewer healthful products on their websites in lower income countries (LICs), while simultaneously highlighting their charitable activities to appeal to consumers in LICs. Interestingly, promotional differences were also identified within countries—across regions and cultures.^40^ McDonald's in India was found to operate two websites, with all (100%) the pages for the rural and historically poorer north containing promotional material aimed at children, while only 13% of the southern Indian pages contained such material.^40^ Such findings highlight the need for policymakers to be aware of the marketing strategies aimed at adolescents in LMICs in order to develop guidelines and regulations that are specific to LMIC settings, particularly as those settings now feature largely on the radar of transnational fast food corporations.^42^

From a public health perspective, the damaging health effects associated with the contribution of fast food consumption to the rise in the global burden of obesity, cannot be over‐stated.^43^ Many health organizations have raised concerns about the significant burden this will place on the stressed health systems of many less industrialized countries.^44^, ^45^ These warnings are strikingly similar to those resulting from the globalization of tobacco marketing, when tobacco companies began to market to developing countries in the 1970s.^46^ Yet, in a similar manner, these warnings have hitherto failed to result in concrete efforts to address the growth of fast food marketing to vulnerable groups in LMICs. Studies have highlighted the lack of data on the marketing strategies used in LMIC settings as a barrier to achieving such progress.^8^, ^47^

Therefore, by bridging the knowledge gap, this review will guide the development of guidelines and regulations that prohibits the marketing of fast foods to adolescents in LMICs. Furthermore, knowledge of the marketing techniques being used in LMICs can empower the public health workforce to utilize similar techniques to promote healthier options to adolescents or reduce the impact of their exposure to unhealthy foods. Consequently, this review seeks to identify the common marketing strategies used by transnational fast food brands when promoting their products to adolescents in LMICs.

## 2 METHODOLOGY

**FIGURE 1:**
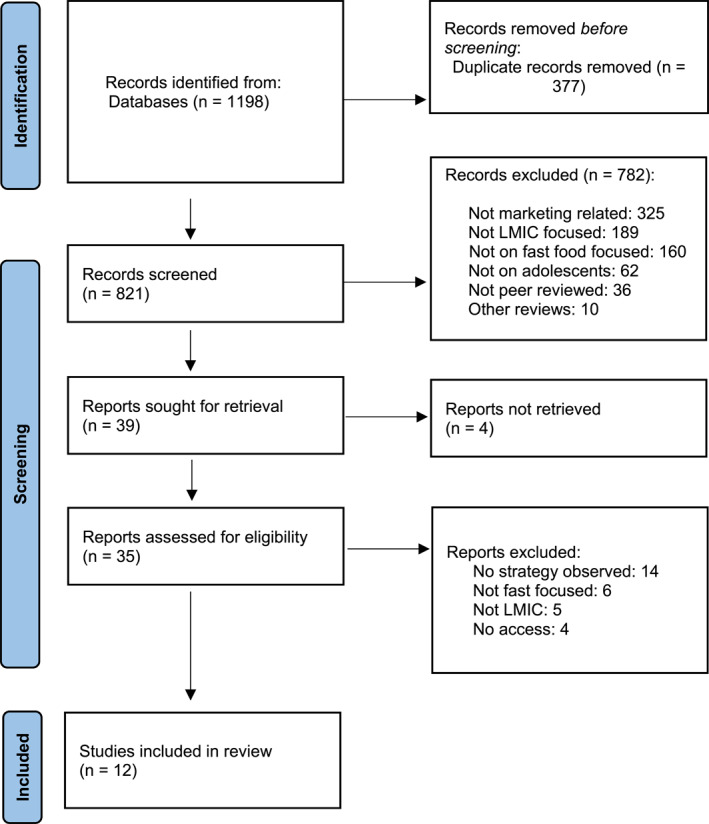
PRISMA 2020 flow diagram of systematic search.

Eight academic databases (PubMed, CINAHL, Medline, Embase, ProQuest, PsycInfo, Scopus and Google Scholar) were searched using the PRISMA review process, the results of which are outlined in Figure 1. The databases were searched with terms and variations of the following: “fast food” OR “convenience food” OR “junk food” AND promotion OR marketing AND adolescent* OR teen* OR youth OR “young adult.”

Primary research articles written in English, and published between 1 January 2010 and 30 December 2022, were included if they documented any activity undertaken by a transnational fast food company to promote fast food to adolescents in LMICs. As depicted in Figure 1, studies were excluded if they were not peer reviewed.

The quality of each included article was assessed using a condensed version of the Consolidated Criteria for Reporting Qualitative Research.^48^ Using this tool, five specific quality questions, relevant to the studies included in this review were identified. These questions were similar to those identified in a similar review.^49^ Consequently, the studies were assessed based on whether (i) study aims were clearly defined; (ii) the samples (transnational fast food corporations) were representative of the population; (iii) study limitations were acknowledged; (iv) a description was provided on how the themes (of marketing strategies/techniques) were identified; and (v) whether the data were analyzed by more than one researcher. In line with Cochrane recommendations, a three‐point scale was used to grade the quality items (1 = *item definitely met*, 2 = *partially met*, 3 = *not met*). Thus, a high‐quality paper could score 5 and a low‐quality paper could score 15.

### 2.1 Synthesis of the literature

A narrative synthesis of the reviewed literature was conducted. Focusing on the most common promotional channels and marketing strategies, the findings of the studies were compared and contrasted to identify similarities and differences. Relationships in the data were also explored, and associations between the study outcomes and other factors related to the study (e.g., study design) were examined. Lastly, reasons for similarities and differences were explored systematically, with possible explanations for the pattern of results provided.

## 3 RESULTS

This review identified 12 articles that met the inclusion and exclusion criteria, these included studies were published between 2015 and 2022. The main study properties, quality assessment scores and the relevant findings of the reviewed articles are presented in Table 1.

**TABLE 1:** Study characteristics and key marketing strategies used to promote fast food to adolescents in LMICs.

Authors (study location)	Title	Study design	Promotional channel	Marketing activities
Guimarães et al., 2022.^50^ (Brazil)	Abusive advertising of food and drink products on Brazilian television	Content analysis	TV adverts	Fast food meals were promoted using children's characters or presenters, animations; and offering prizes and free gifts such as toys that drive excess consumption
Cassidy et al., 2021.^51^ (8 LMICs^a^)	Comparing McDonald's food marketing practices on official Instagram accounts across 15 countries	Content analysis	Instagram	Special price promotions and free giveaway/vouchers on accounts, brand characters
Shabuz & Bexci, 2019.^52^ (Bangladesh)	Dwindling influences of television advertisements on the consumption of branded snacks and beverages	Descriptive study	TV adverts	Celebrity endorsements; advertorials showcasing visuals of their food; and utilizing music, graphics and animations that promote brand familiarity
Vassallo et al., 2018.^53^ (Global)	Junk food marketing on Instagram: Content analysis	Content analysis	Instagram	Posting images of “everyday” people consuming their product
Gaber et al., 2018.^54^ (Egypt)	Why do consumers use Facebook brand pages? A case study of a leading fast‐food brand fan page in Egypt	Content analysis	Facebook	Posting funny memes; incentives like prizes/discounts; and general interactive posts seeking feedback
Jaichuen et al., 2018.^55^ (Thailand)	Food marketing in Facebook to Thai children and youth: An assessment of the efficacy of Thai regulations	Content analysis	Facebook	Posting pictures with a focus on advertising special price promotions/reductions; posting branding elements to increase brand loyalty
Gupta et al., 2017.^5^ (India)	Content of food advertising for young adolescents on television	Content analysis	TV adverts	Advertisements showcasing appealing visuals of their food product
Mazariegos et al., 2016.^8^ (Guatemala)	Nutritional quality and marketing strategies of fast food children's combo meals in Guatemala	Descriptive study	In‐store banners	Toy giveaways; offering price incentives, combo meals and making health claims
Saad & Badran, 2016.^56^ (Egypt)	How successful is fast food social media marketing? International versus Local chains	Content analysis & cross‐sectional survey	Facebook	Offering discount prices if users re‐share posts. Using entertaining posts that encourage the audience to interact or broaden discussions about a topic, product or brand
Harris et al., 2015.^57^ (global)	Marketing unhealthy foods to children on Facebook	Content analysis	Facebook	Posting photos and posts that encourage users to share with a friend or interact with the brand
Amanzadeh et al., 2015.^1^ (El Salvador)	An interpretive study of food, snack and beverage advertisements in rural and urban El Salvador	Visual interpretive study	Billboards and stickers	Promoting products as cheap, economical and modern; on billboards around urban areas and on stickers around rural areas
Freeman et al., 2014.^56^ (Global)	Digital junk: food and beverage marketing on Facebook	Content analysis	Facebook	Offering toys; and posting special price promotions on their pages

Abbreviation: LMICs, low and middle‐income countries.

^a^ 8 LMICs: Romania, Lebanon, Malaysia, Brazil, South Africa, Indonesia, Egypt and India.

### 3.1 Study properties

As shown in Table 1, the included articles were of acceptable quality, with scores ranging between 5 and 11, and a median score of 7. This indicates that the study aims, sample, and methods used to explore the marketing strategies were clearly outlined; coding and inter‐coder reliability and study limitations were assessed and reported. More than half (*n* = 8) of the studies examined fast food marketing in middle‐income countries, with one study^51^ analyzing marketing content across 8 middle‐income countries, 3 lower‐ and 4 upper middle‐income countries.^58^ The remaining articles though conducted in HICs, explored marketing avenues that were accessible to LMICs adolescent population. In terms of research objectives, only two papers measured the effect of the marketing strategies on adolescents.^50^, ^52^ One article compared the social media marketing practices of McDonalds in HICs versus in LMICs, finding more child‐directed themes in LMICs.^51^ Most studies however set out to explore the nature and extent of the marketing strategies used to promote fast food consumption to adolescents in LMICs, even though a few authors had secondary aims which are relevant to the context of this review. For instance, Mazariegos^8^ sought to compare the nutritional quality of combo meals with health claims against those without health claims; while another author^54^ assessed whether a brand's ads complied with the relevant regulation or the industry's self‐regulatory codes of practice.

As seen in Table 1, almost all included articles utilized observational methods, with 10 of the 12 studies consisting of a content analysis of fast food advertisements via television (*n* = 3) or online platforms (*n* = 7). One study^1^ however used a visual interpretive technique to qualitatively describe the marketing strategies of transnational fast food corporations, while another study^8^ assessed the prevalence of commonly used strategies via a quantitative cross‐sectional survey. Two articles^52^, ^55^ utilized mixed method designs to incorporate content analysis and surveys in separate research stages. For example, after analyzing Facebook ads, Saad and Bachran^55^ conducted online interviews with the social media managers to collect relevant information on their brands' marketing strategy.

All of the papers that analyzed online ad content used purposive sampling methods to select the sample of transnational fast food corporations to analyze, with “popularity” being the most common selection criteria. For example, in one case,^59^ the Instagram accounts chosen for analysis were selected based on the number of followers, while another author^54^ selected top 10 fast food brands according to a list made publicly available by Facebook themselves. In contrast, Gupta^5^ who analyzed TV ads, approached school‐going adolescents to identify their 3 favorite channels; while Amanzadeh^1^ and his colleagues chose a convenience sample of food ads based on proximity to rural and urban areas. Overall, McDonalds, KFC and Pizza Hut were the most studied transnational fast food corporations.

In all but two of the studies in this review,^8^, ^54^ deductive methods were used to identify the marketing strategies, with over 20 different strategies identified using coding guides informed by previous research in this area. However, the nature of the documented strategies varied greatly, and were dependent on specific research objectives. For instance, a study interested in the prevalence of industry‐specific techniques focused on transnational fast food corporation's use of health claims, quick delivery claims, toy giveaways and price incentives. Similarly, another study seeking to assess the magnitude and profile of fast food marketing on Facebook, focused on the common food marketing techniques and tactics used on Facebook.^54^ Freeman et al. on the other hand, added two categories to a well‐informed coding tool after piloting the coding tool on the Facebook page of a widely popular food brand that was not part of the study's sample.^55^

### 3.2 Promotional channels

Although some studies were only interested in traditional channels of promotion such as the use of in‐store banners,^8^ billboards^1^ and TV advertisements^5^, ^50^, ^52^: most of the research (*n* = 7) focused on the promotional activities of transnational fast food corporations on social media platforms.^51^, ^53^, ^54^, ^55^, ^56^, ^57^, ^59^ Despite regular reports of the “death” of Facebook^60^, ^61^; the platform still boasts the largest number of global adolescent users, with a potential advertising reach of over 113 million 13–17‐year‐old users.^62^ All but one^53^ of those six articles examined the marketing activities of transnational fast food corporations on Facebook. However, in certain populations, Facebook is less popular than other social media platforms,^63^, ^64^ and the decision of two authors to analyze the Instagram accounts of transnational fast food corporations is perhaps reflective of the ever‐evolving nature of the social media landscape.^51^, ^53^ Overall, the evidence base reflects that social media is the most popular promotional channel, with the combined studies covering over 40,000 social media posts.

### 3.3 Marketing strategies

All of the studies documented various marketing strategies used by trans‐national fast food corporations. To code these strategies, we drew on descriptions of teen‐directed food marketing techniques by Truman et al. and Hebden et al. (Box 1).^24^, ^65^ The results showed that the most common strategies used to promote fast food were the offering of incentives or premium offers (*n* = 6), brand marketing (*n* = 5), product appeal (i.e., when claims about value, health or taste are made about a product, *n* = 4), and promotional characters (*n* = 4).

**Box 1 Codebook of adolescent‐targeted marketing strategies.**

1

**Table:** 

Marketing strategies	Examples	Marketing strategies	Examples
Brand marketing	Product placement, brand influencers	Incentives or premium offers	Giveaways, vouchers, coupons/rebates, price discount and meal deals
Promotional character	Celebrities, branded characters, licensed characters and sports figures	Product appeal	Product design, product appeal/claims
Events/publicity	Philanthropic marketing/corporate social responsibility (CSRs), publicity	Settings‐based tactics	Retail/point‐of‐purchase displays, products in schools/playgrounds
Games and play	Advergames, quizzes/polls	Sponsorship	Sports and other event sponsorship

*Note*: Truman et al.^24^ and Kelly et al.^65^

Two studies examined the effect of the marketing strategies on adolescents. One of those studies assessed the correlation between adolescents' consumption of fast food products and their exposure to TV ads, reporting a negative correlation between both variables.^52^ Alternatively, Gaber and his colleagues measured the effect as the level of consumer engagement by counting the number of likes, comments and shares of a post on the brand page. However, the demographics of the engaged population was not provided.^54^ Although such data exist, access is often limited to the providers of digital platforms making it difficult for researchers to determine whether adolescents are the main target of digital advertisements. Two studies cited this restricted access as a limitation of their study.^55^, ^59^ One study, however, was able to assess the targeted‐marketing activities of transnational fast food corporations by creating Facebook accounts for two hypothetical 13‐year‐old boys and interacting with a fast food brand on the platform.^59^

## 4 DISCUSSION

The evidence base reflects that social media is now commonly used to promote fast food to adolescents in LMICs. Other reviews have suggested TV as the most used medium to reach children and adolescents.^36^ However, TV dominance appears to have waned and in more than 104 countries, young people make up about 80% of the Internet population, with 39% of all young people using the Internet residing in India and China.^35^ The finding of this review reflects the changing patterns of media use among adolescents, even for those in LMICs.

Facebook was the most explored digital platform, with over 35,000 Facebook posts examined by the studies in this review. These posts consisted of pictures, funny memes, videos and quizzes directly posted by the account of transnational fast food companies to promote fast food to their adolescent Facebook fan base. However, since Facebook's popularity is waning among young people all over the world and adolescents now spend more time on other social media apps like Snapchat, TikTok and Instagram,^66^ it would be worthwhile for new studies to examine adolescent exposure to fast food marketing via the social media applications that enjoy more popularity.

Peer‐group influence is crucial on social media, and it is common practice among adolescents to co‐view, share, and discuss media content with peers.^67^, ^68^ Critically, the majority of the brands observed by the studies in this review encouraged users to comment, like or share their posts; as any form of engagement with these posts may appear on the news feed of the users' friends, effortlessly spreading the promotional message across the network. In 2020, one in four Facebook users engaged with a post by a brand/company page, with photo and video posts observed to attract higher levels of engagement.^69^ Indeed, all six social media analyses included in this review found that the bulk of the social media posts used to promote food to adolescents in LMICs consisted of images and videos. Interestingly, one of those studies reported transnational fast food corporations prominent use of hashtags on Facebook—a social media technique not documented in any other analysis—to amplify promotional messages and extend its reach to larger numbers of Thai adolescents.^47^ Multiple reports from Thailand suggest that the application of hashtags is a popular and effective marketing technique in the country.^70^, ^71^ As a result, this finding, though isolated, is proof of the hugely dynamic and contextual nature of food marketing via digital channels.^47^

Offering incentives such as toys, coupons and price discounts to adolescents associated with the purchase of a product; was unsurprisingly the most common marketing strategy used to promote fast food to adolescents in LMICs. Food price has always been a major driver of food choices and although adolescents' spending power tends to increase as they grow older, their finances remain limited, particularly for those in LMICs.^72^ Therefore, the use of special promotions involving price discounts to attract adolescents in LMICs was not an unexpected finding. However, it is interesting to note that apart from the promotion of fast food as a “cheap” calorie source, transnational fast food corporations also promote their products in LMICs, as one that gives young people more value for their money. To that effect, combo meals are created and promoted as a way for children and adolescents to “eat more while spending less.”^8^ This theme (of value‐for‐money) was among the most common persuasive techniques observed in this review. It was however not as common as the use of premium offers like toys and discount vouchers—a technique which is also common in high‐income settings.^49^

In striking contrast to transnational fast food corporations' strategy in high‐income countries, and perhaps because of varying levels of food literacy,^73^ fast food was rarely promoted to these adolescents as healthy food.^40^ One study even reported that basic nutritional information on fast food items was not easily accessible to consumers; in contrast to HICs,^8^ where ads usually tout the nutritional information of fast food to attract customers.^1^ Instead, transnational fast food corporations' ads in LMICs were focused on extoling visual, sensory and satiate elements of their products such as the appearance, taste and preparation time. This review found that such promotion of product elements was a common marketing strategy aimed at adolescents in LMICs—a finding that is likely related to the industry's aim to drive changes in the cultural expectations of food in the developing world.^19^, ^74^

The current generation of adolescents in developing countries are connected both to their counterparts in the developed world and to western media where appealing images and sensations of fast food products are regularly broadcasted.^69^ Therefore, frequent use of similar product appeals in advertisements is likely to stimulate the adolescent's innate interest to try something new, particularly if the “new” food is one that is popular among their peers in western countries. However, it is also possible that the promotion of product elements is also aimed at capitalizing on the consumer culture in LMICs.^75^

Global food brands are often attracted to the new and emerging markets of LMICs because of a consumer culture characterized by the desire for goods and services which are in consonance with international standards.^4^, ^76^ By highlighting key product elements, transnational fast food corporations' can ensure consumers that their products and services are not different from what is on offer in developed countries.^76^ Additionally, consumers are believed to make logical and rational decisions about products based on product benefits.^77^ By extoling the ultra‐modified characteristics of fast food like its taste and appearance; transnational fast food corporations are appealing to potential consumers to make the “rational” decision to eat fast food because it tastes and/or looks good. Indeed, the literature confirms that such palatable cues increase the rational appeal for unhealthy food especially for teens,^78^ and so approaches such as the modification of taste remain central to the globalization of the food industry.^79^, ^80^

Food marketing seeks to change consumption habits over the long term.^19^ To ensure lifelong purchases, developing brand loyalty at a young age is fundamental.^3^ Therefore, it was no surprise that strategies aiming to increase brand familiarity, were among the most common strategies observed in this review. With such huge global recognition, marketing by transnational fast food corporations involves leveraging their brand power.^22^, ^81^ Strategies aimed at increasing brand familiarity among adolescents include the use of endorsers or influencers.^24^ In LMICs though, fast food was portrayed as popular not only among celebrities but also among members of the general public, with one study observing promotional posts showing “everyday” people consuming fast food. Contrary to expectations, the transnational fast food corporations observed in this review rarely advertised their philanthropic activities while promoting fast food to adolescents in LMICs. Previous studies have shown that global fast food brands publicize their CSR and/or philanthropic activities to build consumer's loyalty to their brand.^22^ It is however possible that this discrepancy is due to our focus on strategies targeting adolescents, with young people unlikely to be perturbed about social issues such as charity and donations.

To the best of our knowledge, this is the first systematic review to examine the marketing strategies used to promote fast food to adolescents in LMICs. Other recent systematic reviews of food advertising to adolescents have focused on specific channels such as TV ads,^49^ despite the increasing range of promotional channels used to target adolescents. A key strength of this review is that we examined adolescent's exposure to fast food marketing via all documented channels of exposure. Therefore, in terms of generalizability of the common marketing strategies, our deductions can be taken to represent marketing strategies across all media. In addition, by using a broad range of adolescent‐specific marketing strategies as coding guide,^24^, ^65^ this review is useful for effective detection and monitoring of fast food marketing to adolescents in LMICs, in order to inform policy and advocacy in these settings.

Examining exposure on platforms as dynamic as social media presents new methodological challenges for researchers.^47^ Generally, advertising via any digital media is tailored either to the content user views on the platform (contextual advertising) or to the characteristics and preferences of the user (behavioral advertising). In terms of the latter, information developed on users is used to create individual profiles that allow brands to use specific targeting and marketing approaches.^47^ For example, corporations rely on geo‐location data from mobile devices to deliver ads and special offers in real time when users are in the location where they are sold.^47^, ^82^ On Facebook, these customized and targeted ads will show up within the “sponsored” column of the user's news feed, therefore, solely monitoring and documenting on the general Facebook posts of the food brand does not reveal the full extent of a brand's marketing activity. For customized and targeted ads to be observed, public health researchers need to create hypothetical social media accounts within the target location, and then interact with the food companies' page.^47^, ^83^ A similar approach was conducted in only two of the studies included in this review.^41^, ^59^ Therefore, there is a chance that adolescents' exposure to fast food marketing on digital channels has been underestimated. This could be regarded as a limitation of this review.

Globally, the price of a product is a major influencer of purchase decisions among all age groups.^84^, ^85^ However, consumers in many LMICs, attracted to the glamourized modernity and status symbol that patronizing transnational fast food corporations represents, are usually willing to pay a relatively higher price for fast food meals.^85^ Therefore, in terms of policy implications, although this review does not examine the marketing strategies of local food vendors—a topic worthy of further research‐the frequent use of price discounts and vouchers by transnational fast food corporations suggests that price is still an important driver of adolescents' food choices in LMICs. For policy and decision‐makers, this is crucial information, as one of the most common interventions to reduce the consumption of unhealthy commodities involves taxation and other financial mechanisms that indirectly lead to an increase in the price of these products. In that regard, the findings of this review seem to suggest that the implementation of legislation restricting transnational fast food corporations from offering financial incentives to adolescent or even all consumers, might be an effective policy intervention to reduce fast food consumption rates among adolescents.

As noted previously, social media applications are rapidly becoming the preferred medium to market fast foods to adolescents. It is estimated that adolescents view over 9000 food marketing ads per year on social media apps.^86^ Yet, the policies and legislations in the few LMICs that have taken action, are focused on TV advertisements and other traditional media,^8^ despite recent findings suggesting that digital marketing could be more effective than traditional marketing.^87^, ^88^ Given the transnational fast food corporations preference for digital media and the pervasive nature of the marketing strategies found on digital platforms, this review emphasizes the need for proactive consideration and implementation of strict regulations to manage fast food marketing within digital environments.

Lastly, understanding the target market is crucial to maximize the effectiveness of global food marketing.^24^ This review found contextual differences between the marketing strategies identified including in how they were deployed, supporting the literature that transnational fast food corporations adjust marketing activities based on the results of market research which they significantly invest in ‐and rely on‐, to understand consumer habits, likes and dislikes, preferences and prejudices.^89^ This further suggests that to “compete” with “Big Food,” health promoters seeking to promote healthy food options also need to evolve their marketing activities by comprehensively defining and researching the target market.

## 5 CONCLUSION

This review documented the most common marketing strategies used to promote fast food to adolescents in LMICs. Contrary to the literature around fast food marketing in LMICs, social media is more commonly used to reach adolescents than any other promotional channel. Global fast food firms interact with adolescents living in LMICs via the wide range of tools which social media platforms provide, driving the promotion using digital techniques that could differ based on contextual social media characteristics. Due to such dynamism, it could be suggested that adolescent‐directed fast food marketing on social media is best observed locally and inductively to monitor and restrict adolescent exposure to fast food advertisements. However, peers exert influence on adolescents via user‐generated content on social media, therefore new studies need to adopt methodologies that allow such exposure to be examined. Despite research suggesting that consumers in LMICs are not deterred by the relatively high cost of fast food, this review shows promise that restricting transnational fast food corporations from offering price discounts and vouchers to adolescents could significantly impact consumption rates.

## CONFLICT OF INTEREST STATEMENT

There are no conflicts of interest to disclose.
